# Untargeted Metabolomics Studies of H9c2 Cardiac Cells Submitted to Oxidative Stress, β-Adrenergic Stimulation and Doxorubicin Treatment: Investigation of Cardiac Biomarkers

**DOI:** 10.3389/fmolb.2022.898742

**Published:** 2022-06-29

**Authors:** Monica Força Lima, Alan Gonçalves Amaral, Isabela Aparecida Moretto, Franckson Jhonne Torres Neves Paiva-Silva, Flávia Oliveira Borges Pereira, Coral Barbas, Aline Mara dos Santos, Ana Valéria Colnaghi Simionato, Francisco Javier Rupérez

**Affiliations:** ^1^ Center for Metabolomics and Bioanalysis (CEMBIO), Facultad de Farmacia, Universidad San Pablo-CEU, CEU Universities, Madrid, Spain; ^2^ Department of Analytical Chemistry, Institute of Chemistry, University of Campinas (UNICAMP), Campinas, Brazil; ^3^ Department of Structural and Functional Biology, Institute of Biology, University of Campinas (UNICAMP), Campinas, Brazil; ^4^ National Institute of Science and Technology in Bioanalytics (INCTBio), Campinas, Brazil

**Keywords:** cardiac hypertrophy, oxidative stress, metabolomic analysis, CE-MS, H9c2 cells

## Abstract

One of the biggest challenges in the search for more effective treatments for diseases is understanding their etiology. Cardiovascular diseases (CVD) are an important example of this, given the high number of deaths annually. Oxidative stress (the imbalance between oxidant and antioxidant species in biological system) is one of the factors responsible for CVD occurrence, demanding extensive investigation. Excess of reactive oxygen species (ROS) are primarily responsible for this condition, and clinical and scientific literature have reported a significant increase in ROS when therapeutic drugs, such as doxorubicin and isoproterenol, are administered. In this context, the aim of this study is the investigation of potential biomarkers that might be associated with oxidative stress in cardiomyocytes. For this purpose, H9c2 cardiomyocytes were submitted to oxidative stress conditions by treatment with doxorubicin (DOX), isoproterenol (ISO) and hydrogen peroxide (PER). Metabolomics analyses of the cell extract and the supernatant obtained from the culture medium were then evaluated by CE-ESI(+)-TOF-MS. Following signal processing, statistical analyses, and molecular features annotations, the results indicate changes in the aspartate, serine, pantothenic acid, glycerophosphocholine and glutathione metabolism in the cell extract.

## 1 Introduction

Oxidative stress is a biological condition known to alter the appropriate working of different biological structures, from morphological levels to modifications of cellular metabolism. Such state is stablished when an imbalance between the production of oxidant species (such as reactive oxygen species - ROS, and reactive nitrogen species - RNS) and the presence of produced or ingested antioxidant species occurs in the system (Sies et al., 1998; Senoner and Dichtl, 2019; Tejero et al., 2019). Oxidative stress has been related to the pathogenesis of several diseases, including cardiovascular diseases (CVDs) (Rodrigo et al., 2013; Senoner and Dichtl, 2019). Hydrogen peroxide (H_2_O_2_) is a non-radical ROS, which despite not being highly reactive and presenting a low oxidizing power, it is a precursor of very reactive and oxidizing species, such as the hydroxyl radical in the presence of Fe^2+^ or Cu^+^ metal ions, as presented and proposed in the Fenton reaction (Meneghini, 1997; Charles and Eaton, 2008; Ayala et al., 2014). Despite the natural occurrences that can lead to oxidative stress, such as poor diet, reckless physical exercises, and diseases, several studies have indicated an increased ROS production when some therapeutic drugs are administered to patients. Among them, isoproterenol, used in the treatment of cardiac arrhythmias and bronchospasm, and doxorubicin, used for the treatment of some types of cancer, can be highlighted (Kondo et al., 1987; Octavia et al., 2012).

Isoproterenol, a β-adrenergic agonist and a synthetic catecholamine, is primarily used in the treatment of bradycardia, TIP (Treatment Improvement Protocols) disorders and heart blockage (Kemper et al., 1983; McMahon et al., 2011). It causes cardiac tissue (cardiomyocytes) oxidative stress due to catecholamine auto-oxidation, which stimulates lipid peroxidation, an irreversible myocardial membrane damage (Zhou et al., 2008; Octavia et al., 2012). Isoproterenol is widely employed in cardiovascular research as an adrenergic stimulation inducer in the study of protective and preventive effects against severe cardiac dysfunctions (Wexler and Judd, 1970; Wexler, 1978; Zhang et al., 2009). Sun et al., Zhang et al. and Liu et al. developed LC-MS methods for the analyses of metabolites in rat blood samples following isoproterenol treatment (Zhang et al., 2009; Liu et al., 2014; Sun et al., 2017). Sun and col. identified 16 oxidative stress biomarkers, including nitric oxide, taurine, and those related to hypotaurine and sphingolipids metabolisms (Yi et al., 2018). Zhang et al. identified 13 lipid species, including Lyso-PCs and fatty acids, as potential biomarkers in the blood of isoproterenol-treated rats (Zhang et al., 2009), while Liu and col. detected 8 biomarkers related to myocardial infarction induced by isoproterenol (Liu et al., 2014), involved in the beta-oxidation of fatty acids and in the sphingolipid metabolism, proteolysis, tryptophan and purine metabolisms.

Doxorubicin is a drug used to treat certain types of cancer, although it may cause cardiotoxicity, among other damage, and often leads to congestive heart failure (Tan et al., 2011; Octavia et al., 2012). Several studies have attributed doxorubicin-induced cardiomyopathy to increased free radical formation, lipid peroxidation, cardiomyocyte apoptosis, interference in calcium dynamics, mitochondrial abnormalities, irreversible DNA damage, among others. Tan et al. and Chen et al. developed methods employing LC-MS and GC-MS to investigate myocardial tissue effects caused by doxorubicin, searching for possible biomarkers (Tan et al., 2011; Chen et al., 2015). Tan and col. reported that six metabolites (citrate, isoleucine, threonine and others) exhibited decreased levels in hearts from DOX-treated mice (Tan et al., 2011). Yi et al. developed a method using LC-MS to characterize metabolic changes in H9c2 cells undergoing DOX treatment and subsequently treated with Danhong injections, a Chinese traditional medicine. This study revealed amino acid and nucleotide metabolism alterations (Yi et al., 2018). Despite scientific literature reporting information on the impacts of excessive ROS production due to isoproterenol and doxorubicin treatment, further investigations are still required in order to better comprehend the connection between oxidative stress and related diseases. For instance, Amaral et al. have recently developed an untargeted metabolomic investigation by LC-MS to assess the effect of oxidative stress on H9c2 cells (cardiomyocytes) using H_2_O_2_. After 24 h of treatment, they verified changes in the metabolism of alanine, aspartate, glutamate and glutathione, in addition to changes in glycolysis, pointing out to possible diagnostic biomarkers (Amaral et al., 2022).

In this context, biomarkers, defined by Food and Drug Administration (FDA) as “an objective measure that acts as an indicator of normal biological processes, pathogenic processes or pharmacological responses to a therapeutic intervention”, may comprise an important tool to obtain a disease diagnosis (Wagner and Atkinson, 2015). An ideal biomarker must present adequate specificity and sensitivity to distinguish the disease establishment between a healthy (control) group and an affected group (Turner et al., 2009). Metabolomics approaches have been previously employed in the investigation of heart failure biomarkers. Lai et al. reported increased acylcarnitine levels and decreased TCA cycle intermediate metabolites levels in rat models with induced heart failure compared to hypertrophic rat models (Lai et al., 2014). Aubert et al. and Bedi et al. verified decreased cardiac fatty acid oxidation and increased ketone oxidation in rat models with heart failure (Aubert et al., 2016; Bedi et al., 2016), while Lewis et al. identified changes in the circulation of metabolite levels that participate in the pyrimidine metabolism, the tricarboxylic acid cycle and the pentose phosphate pathway in patients who underwent a planned myocardial infarction (Lewis et al., 2008). 

Due to the complexity of biological samples, a combination of different analytical techniques, i.e., LC-MS, GC-MS, and CE-MS are employed to ensure complete biological system information (Álvarez-Sánchez et al., 2010; Zhang and Ramautar, 2021). Of particular interest, CE-MS offers alternative capabilities to the identification of polar, hydrophilic compounds and has not been previously applied to investigate the afore mentioned models and treatments. The aim of this study was to perform an untargeted metabolomics investigation in differentiated heart cells of the H9c2 cardiomyocytes lineage cultured *in vitro*, submitted to oxidative stress conditions through exposure to hydrogen peroxide (PER), beta-adrenergic stimulation using isoproterenol (ISO) and the doxorubicin treatment (DOX) by capillary electrophoresis (CE) coupled to mass spectrometry (MS) to assess the various mechanisms involved in the development of macro (morphology) and micro (metabolism) modifications in a biological system which mimics *in vivo* heart.

## 2 Materials and Methods

### 2.1 H9c2 Cell Culture

H9c2 cells (cardiomyoblasts) were obtained commercially (AddexBio C0031002) and cultured in DMEM medium (Sigma-Aldrich), supplemented with 2 mmol L^−1^ L-glutamine, 1 mmol L^−1^ sodium pyruvate, 1500 mg L^−1^ sodium bicarbonate, 1.0 g L^−1^ glucose and 10% fetal bovine serum (Gibco) in glass plates (Pyrex). 10^6^ cells were plated and after reaching 80% confluence, differentiation was induced for 7 days by the reduction of fetal bovine serum to 1% and the addition of 1 μmol L^−1^ of retinoic acid (Sigma-Aldrich) to the culture medium. The differentiation medium was changed every 2 days (Pereira et al., 2011; Suhaeri et al., 2015; Patten et al., 2017). The cells (cardiomyocytes) were then separated into three groups treated with 100 μmol L^−1^ H_2_O_2_ (Merck) for 1 h (Oraki Kohshour et al., 2013; Ilavenil et al., 2015), 10 μmol L^−1^ of isoproterenol (Sigma-Aldrich) for 48 h (Chen et al., 2012), and 1 μmol L^−1^ of doxorubicin (Sigma-Aldrich) for 12 h (Jiang et al., 2020). This treatment was performed in triplicate for each condition and a control was performed without the addition of any compound (all drugs were prepared in water). For the immunofluorescence assays, H9c2 cells were fixed with 4% paraformaldehyde, blocked and permeabilized with 3% bovine serum albumin and 0.1% Triton-X in 0.1 mol L^−1^ DPBS on ice. Then, control cells and those treated with doxorubicin or H_2_O_2_ were incubated with anti-γ-H2AX (1:200) primary antibodies for 30 min at room temperature. Cells were labeled with Alexa Fluor-488-conjugated goat anti-mouse (1:2,000) and Alexa Fluor-647 phalloidin for 30 min at room temperature. Cells treated with isoproterenol were incubated only with Alexa Fluor-647 phalloidin. Slides were then mounted with Prolong with DAPI. Fluorescence images were acquired on a Zeiss Elyra PS.1 microscope. Panoramic images were examined by an EC Plan-Neofluar 10x/0.30 objective in the laser WideField mode. “Zoomed in” images were collected by an Plan-Apochromat 63x/1.4 Oil DIC objective in the 3D-SIM mode. 3D-SIM z-stacks were projected on a single plane with summed intensities using the ImageJ-FIJI software.

### 2.2 Sample Preparation

#### 2.2.1 Cell Extraction

After each cell treatment, the supernatants were collected and 800 µL of cold methanol were added to the cell flask. Then cells were slightly detached with a rubber tip cell scraper. Samples were transferred to sealed microtubes and stirred vigorously by vortexing. Samples were subjected to three freeze-thaw cycles for complete cell disruption, in order to extract endo-metabolites. For this purpose, samples were placed in liquid nitrogen for 10 min for rapid freezing, and thawed in an ice bath for 10 min. Then, samples were vigorously stirred by vortexing and centrifuged at 5,725 × *g* for 5 min (at 4°C). The supernatants were subsequently transferred to new microtubes. The pellets were re-extracted with 400 µL of cold methanol and, finally, all supernatants were combined and stored at -80°C until analysis. Methanolic extracts obtained from cells were thawed on ice and shaken vigorously by vortexing. A total of 60 µL of each sample were then added to 1.5 mL microtubes and evaporated in Speed Vac Concentrator (Thermo Fisher Scientific, Waltham, MA) at 35°C. Next, 60 µL of an aqueous solution containing 0.1 mol L^−1^ of formic acid (Sigma-Aldrich) and 0.2 mmol L^−1^ of methionine sulfone (Sigma-Aldrich) were added and the mixtures were stirred for 1 min. Samples were finally transferred to a 30 kDa porosity Centrifree^®^ Ultrafiltration Device (Millipore Ireland Ltd., Cork, Ireland), centrifuged for 80 min at 400 x *g* (at 4°C) (Dettmer et al., 2011; Mastrangelo et al., 2016), and the obtained filtrates were added to CE polypropylene vials (Agilent Technologies, Waldbronn, Germany). Quality control samples (QC) preparations were carried out by adding 5 µL of each sample to a single vial.

#### 2.2.2 Culture Medium Examination

The supernatants obtained from each culture medium were thawed on ice and then shaken vigorously by vortexing. A total of 200 µL of each supernatant were then transferred into 1.5 mL microtubes and mixed with 5 µL acetonitrile, 5 µL formic acid (4.1 mol L^−1^) and 5 µL methionine sulfone (22.5 mmol L^−1^). Samples were subsequently vortexed for 1 min, transferred to a 30 kDa porosity Millipore filter and centrifuged for 80 min at 400 × g at 4°C (Villaseñor et al., 2019). The obtained filtrates were added to CE polypropylene vials. The QCs were prepared by adding 16.7 µL of each sample to a single vial.

### 2.3 Instrumental Analysis

An Agilent 7100 CE equipment coupled to a 6230 TOF/MS mass analyzer was employed for the cell extract analysis, and a 7100 CE equipment coupled to a 6224 TOF/MS mass analyzer was used for the culture medium (CM) analysis. Separation was carried out using a fused-silica capillary (total length of 100 cm; i. d. of 50 μm; Agilent Technologies). The capillary was pre-conditioned using 1 mol L^−1^ NaOH for 30 min, followed by ultrapure water obtained from a Water Milli-Q 185 Plus system (Millipore, Bedford, United States) and background electrolyte (BGE), consisting of 800 mmol L^−1^ of formic acid in 10% methanol, for 30 min, at 20°C, using normal polarity. Before each analysis, the capillary was flushed for 5 min (950 mbar pressure) with BGE. The MS operated in positive polarity, in full scan mode from 70 to 1,000 m/z at a rate of 1.36 scans/s. Drying gas (nitrogen) was set to 10 L min^−1^, nebulizer gas (nitrogen) to 10 psi, voltage to 3.5 kV, fragmentor to 125 V, gas temperature to 200°C and skimmer to 65 V. Sheath liquid composition was methanol/water (1:1 v/v) containing 1.0 mmol L^−1^ of formic acid and two analytical standards for mass references, which allow for mass correction and higher mass accuracy, namely m/z 121.0509 (purine) and m/z 922.0098 (HP-921, a commercial standard). Samples were injected at 50 mbar for 50 s and stacked by applying background electrolyte at 100 mbar for 20 s. The separation voltage was 30 kV with 25 mbar of internal pressure and a total running time of 30 min.

### 2.4 Data Processing

The data acquired by CE-MS were processed using the MassHunter Profinder software (B.07.00, Agilent Technologies, RRID:SCR_017026) to obtain a structured data matrix in an appropriate format. Raw data were analyzed by two algorithms applied consecutively. The first one comprised the molecular feature extraction (MFE) algorithm, which reduces the data size and complexity by removing associated non-specific information and extracting important variables (molecular features), “while second one consisted on the find by ion (FbI) algorithm, used to perform a targeted feature extraction to obtain better data accuracy. Finally, the molecule abundances, masses and retention times for each feature in all samples were obtained in a matrix data form. The quality of the CE-MS data was assured by excluding background noises and unrelated ions by maintaining molecular features present in 50% of QC injections presenting a coefficient of variation (CV) below 20%, and present in 75% of the samples. Then, the obtained data matrix was submitted to a statistical analysis using the Metaboanalyst 4.0 software (www.metaboanalyst.ca) (Chong et al., 2019). Missing values were estimated using the k-nearest neighbors (kNN) algorithm. Samples corresponding to the cell extracts were subjected to sum normalization, logarithmic transformation and Pareto scaling. Cell culture medium was subjected to median normalization, logarithmic transformation and Pareto scaling.

### 2.5 Metabolite Annotation

Annotations were performed for all significant compounds, according to variable importance projection (VIP) values higher than 1.0 and correlations (pCorr) higher than 0.5 by searching the exact masses in the public online databases KEGG (RRID:SCR_012773), LipidMaps (RRID:SCR_003817) and HMDB (RRID:SCR_007712), using the CEU Mass Mediator online tool (Gil de la Fuente et al., 2018). In addition, a pathway analysis for the main comparisons was performed using the MetaboAnalyst 4.0 (RRID:SCR_015539) software.

### 2.6 Statistical Analyses

Data were analyzed by multivariate (MVA) and univariate (UVA) analyses using the Metaboanalyst 4.0 software. For the MVA, both the cell extracts and culture medium supernatants were subjected to normalization by the sum, logarithmic transformation, and Pareto scaling. For the unsupervised modeling, a principal component analysis (PCA) was used to confirm data quality, detect discrepancies and verify sample patterns. In addition, discriminant models, such as the partial least squares discriminant analysis (PLS-DA) and orthogonal PLS-DA discriminant analysis (OPLS-DA) were performed to detect potential group separations. For the UVA, the Mann-Whitney *U* test was initially used to compare the averages and obtain potentially significant characteristics for group discrimination. The level of statistical significance was established at a 95% confidence interval (*p* < 0.05). The Kruskal–Wallis test was then applied. The fold change (FC) was determined using the ratio of average value in the case group/average value in the control, or reference group. The percentage of variation (%) was calculated as follows [(average value in the case group - average value in the reference group)/(average value in the reference group)] × 100.

## 3 Results

### 3.1 Morphological Assessment

**FIGURE 1:**
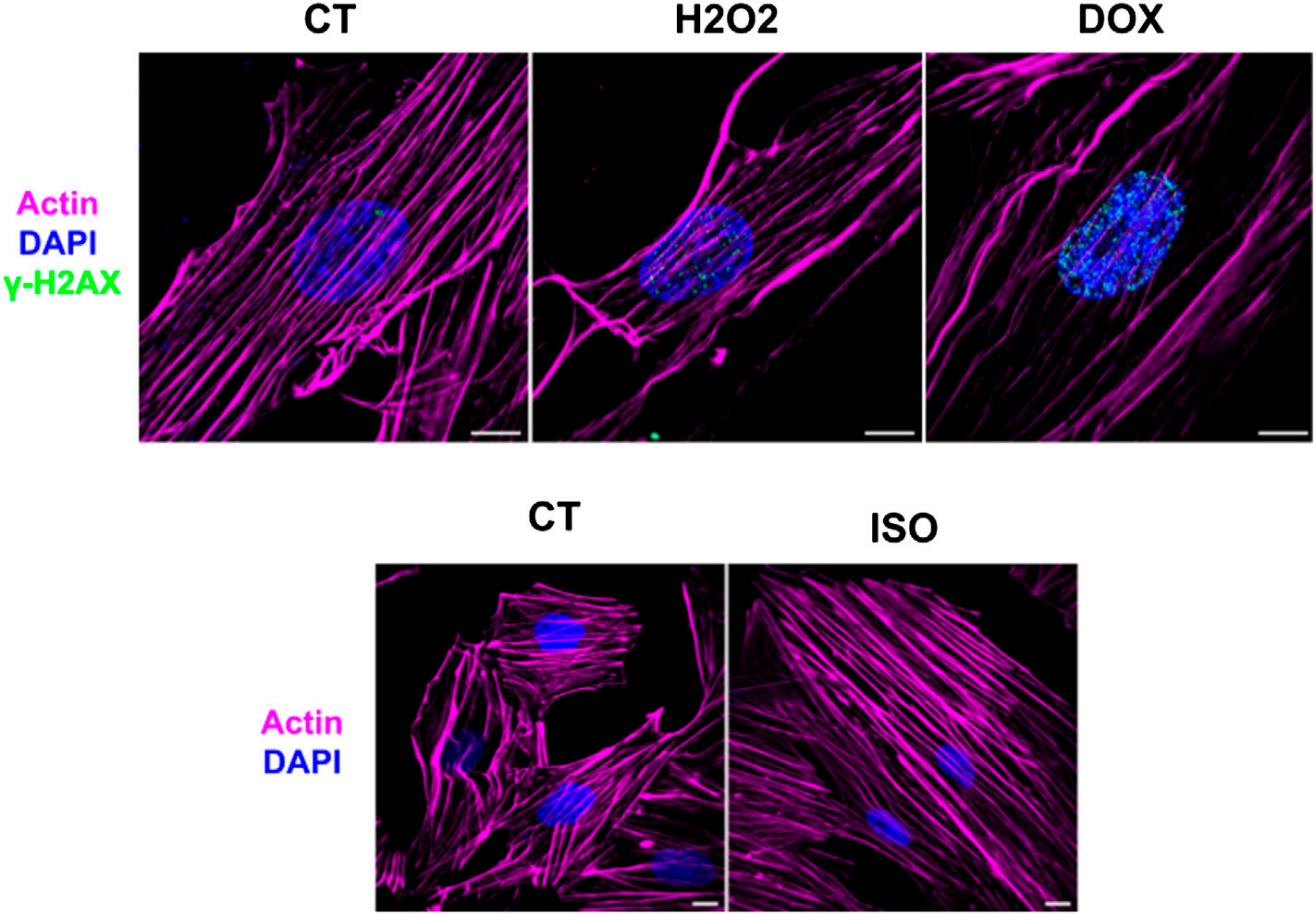
Super-resolution images from control (CT), H_2_O_2_ doxorubicin (DOX) and isoproterenol (ISO) treated H9c2 cardiomyocytes. The efficacy of the oxidative stress resulting from H_2_O_2_ and dox treatment was demonstrated by the increase of the γ-H2AX loci and the effect of isoproterenol was attested by the hypertrophy phenotype of H9c2 cells. Magenta: actin; Blue: nucleus; Cyano: γ-H2AX. Scale bar = 10 µm.

H9c2 cardiomyoblasts were grown in DMEM culture medium for 2 days in Petri plates, and then the differentiation was stimulated by reducing FBS concentration and supplementing with 10 nmol L^−1^ RA. After 4 days in the differentiation medium, cells became elongated, and the differentiation progressed to day 7, when the cells were extracted (Supplementary Figure S1). The immunofluorescence microscopy analysis confirmed that the shapes of the H9c2 cardiomyocytes cells were elongated and partially polynuclear, while the undifferentiated H9c2 cells had spindle-like shapes and were mono-nucleated (Supplementary Figure S2). After the differentiation process, cells were exposed to H_2_O_2_, DOX and ISO compounds and were analyzed by immunofluorescence microscopy to confirm the efficacy of these stress-induced agents (Figure 1). H_2_O_2_ and DOX treatments promoted DNA breaks, as demonstrated by the increase of the γ-H2AX loci marker, a result of the oxidative stress on DNA. Furthermore, the effectiveness of ISO treatment in H9c2 was attested by the hypertrophy phenotype developed after 48 h of treatment.

### 3.2 Quality of the Cell Extract and Culture Medium Supernatant Data

**FIGURE 2:**
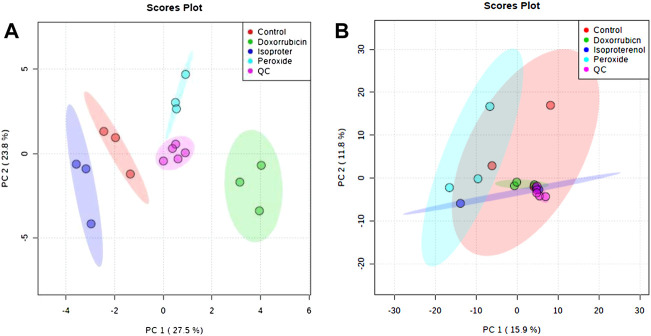
PCA plots generated using all samples and QC analyses by CE-MS in the positive ionization mode. **(A)** Extract obtained from cells and **(B)** Supernatant obtained from cell culture medium.

Supplementary Figure S3 illustrates the obtained cell extract electropherograms (TIE–total ion electropherogram). After data processing, the number of molecular features measured in the cell extracts and in the culture medium supernatants was 81 and 253, respectively. The data quality was verified by a multivariate analysis (MVA) using an unsupervised model. A QCs injection grouping in the PCA space, showing a clustering in the center of the models without prior sample information, demonstrates high data quality (Figure 2).

### 3.3 Metabolic Profile and Statistical Analyses

**FIGURE 3:**
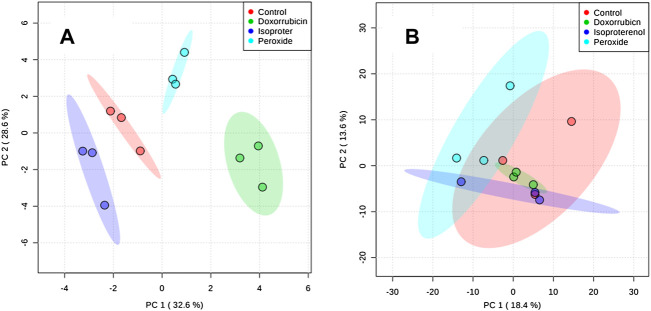
PCA plots generated using all samples. **(A)** Extract obtained from cells and **(B)** Supernatant obtained from cell culture medium.

Another PCA model was developed following QC removal (Figure 3) for both the cell extracts and culture supernatant samples. For the cell extracts, a separation among samples treated with PER, ISO and DOX was noticed in comparison to the control condition. This data suggests that the different treatments distinctively modulate the metabolism of H9c2 cardiomyocytes. However, the culture supernatant did not present differences within any treatment conditions and the control group.

**FIGURE 4:**
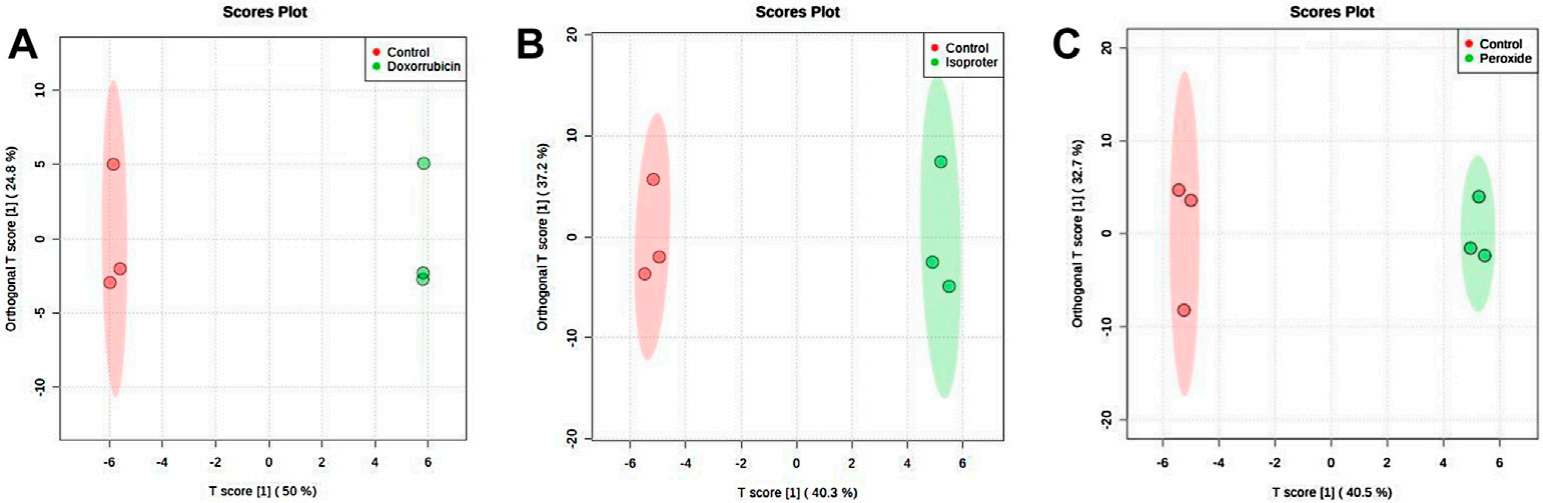
OPLS-DA score plot of all molecular features. **(A)** DOX versus Control, **(B)** ISO versus Control and **(C)** PER versus Control of the cell extract analyzed by CE-(ESI)-MS, in positive ionization mode.

In addition, cell extracts also exhibited differences among groups as verified by both discriminant analysis models (PLS-DA and OPLS-DA) among the pairs of the groups. Figure 4 displays the OPLS-DA validated cross plots validated for the PER, ISO, DOX and control groups. All models displayed a high predictive capacity, with Q2 values above 0.8. Important variables were selected by combining the VIP score (VIP >1.0) and the correlation values (pCorr >0.5) (Table 1).

**TABLE 1:** Significant variables according to VIP and pCorr values of the OPLS-DA model obtained from H9c2 cells extract in the untargeted metabolomic evaluation by CE-MS carried out in the positive ionization mode. FC = Fold Change.

*Mass*	*TM*	*VIP*	*pCorr*	*FC*	*Log*_*2*_ *FC*	Variation (%)	Annotation (Tolerance: 10 ppm)
DOX vs. Control							
103.1000	9.39	1.3307	0.8919	1.0156	0.0222	1.55	Choline
105.0428	12.53	1.7181	0.9871	1.1892	0.2500	18.92	Serine
133.0372	13.87	2.4335	0.9538	1.9592	0.9702	95.92	Aspartic Acid
187.1678	8.90	2.2800	0.9887	1.7147	0.7779	71.47	N-Acetylspermidine
197.0901	20.91	1.0356	0.7048	0.9383	-0,0918	-6.16	Unkown
214.0096	21.94	1.7441	0.8858	1.2369	0,3067	23.69	Unkown
219.1115	20.98	1.2695	0.9844	0.9522	-0.0706	-4.78	Pantothenic acid
241.0927	20.98	1.3649	0.9882	0.9922	-0,0113	-0.78	Methocarbamol
257.1035	20.77	2.1318	0.9712	1.5759	0.6562	57.59	Glycerophosphocholine
264.1044	9.19	1.0749	0.6861	0.9464	-0.0795	-5.36	Thiamine
347.0632	19.82	1.2287	0.6957	1.0542	0,0761	5.42	Adenosine 5-monophosphate
**ISO vs. Control**							
187.1678	8.90	2.0282	0.9463	1.5360	0.6192	53.60	N-Acetylspermidine
257.1035	20.77	1.5514	0.8745	1.3101	0.3896	31.01	Glycerophosphocholine
289.0737	14.22	1,3871	0,8335	0.9435	0,3118	24.12	Unknown
307.0840	15.25	1.4609	0.8983	1.2463	0.3176	24.63	Reduced glutathione (GSH)
337.0947	15.45	1.6191	0.9272	1.3049	0.3839	30.49	S-(Hydroxymethyl)glutathione
612.1537	14.47	1.6416	0.9086	1.3396	0.4218	33.96	Oxidized glutathione (GSSG)
663.1102	20.97	1.4816	0.9885	1.2313	0.3001	23.13	Nicotinamide adenine dinucleotide (NAD^+^)
**PER vs. Control**							
103.1000	9.39	1.5998	0.9728	1.1995	0.2624	19.95	Choline
105.0428	12.53	2.5559	0.9964	1.8713	0.9040	87.12	Serine
133.0372	13.87	2.9346	0.9522	2.4114	1.2698	141.14	Aspartic Acid
146.0690	13.23	1.3922	0.9406	1.1242	0.1689	12.42	Glutamine

The results of the univariate statistical analysis (UVA) using the non-parametric Mann-Whitney *U* tests and the Kruskall-Wallis test did not identify significant molecular features for the evaluated groups. However, the comparison between the pairs of groups using the criterion of *p* < 0.05 and FC higher than 2 or less than −2 by the Volcano plot projection indicates some changes (except for the ISO group, in which no changes were observed). Supplementary Figure S4 indicates the direction of the changes when the DOX and PER pairs were compared to the control.

### 3.4 Metabolic Identification and Pathway Analysis

The list of putatively identified metabolites is resulting in Table 1, totaling 22 metabolites. Amino acids, vitamins, tripeptides and derivatives, carboxylic acids, and polyamines comprise the main metabolite classes. In addition, the variation of each significant metabolite in relation to the control group is exhibited in Table 1 and is presented as the logarithmic function in base 2 of the fold change (Log_2_FC). In general, an increase in metabolite levels compared to the control group was observed for the three evaluated groups. Consistent changes reveal alterations in the aspartate, serine and glycerophosphocholine metabolism. Furthermore, an influence on pantothenate, CoA and arginine biosynthesis were also observed.

The use of doxorubicin led to approximately 95% and 71% increases in aspartate and N-acetylspermidine levels (Table 1), respectively, when compared to the control group, while pantothenic acid and thiamine decreased in this group to 4.8 and 5.4%, respectively. When H9c2 cells were treated with isoproterenol, increased levels of N-acetylspermidine (53.6%) and glycerophosphocholine (31%) levels were also observed, following the same pattern as in the treatment with doxorubicin. Increases in glutathione (and its derivatives), nicotinamide, and adenine dinucleotide (NAD^+^) were also noticed. Increases in choline (19%), aspartate (141%) and serine (87%) were observed in cells treated with hydrogen peroxide when compared to the control. Based on the identified molecular features, the altered pathways were constructed with the pathway analysis tool of the MetaboAnalyst software (Supplementary Figure S5 and Table 2). Results confirm that the most impacted pathways were the alanine, aspartate, glutamate and glutathione metabolisms. The metabolism of alanine, aspartate, and glutamate was shown to be the most impacted pathway upon DOX and PER administration, while glutathione metabolism was the most altered pathway upon ISO administration.

**TABLE 2:** Pathway Analysis Parameters Comparisons - Level of correspondence, negative *p*-value logarithm and impact obtained from the pathway analysis of H9c2 cell extracts.

Group		DOX vs. Control			ISO vs. Control			Per vs. Control		
N^°^	Pathway Name	Match Status	-log(p)	Impact	Match Status	−log(p)	Impact	Match Status	−log(p)	Impact
1	Pantothenate and CoA biosynthesis	2/19	5.0527	0.00714	-	-	-	1/19	2.7873	0.0
2	Alanine, aspartate and glutamate metabolism	2/28	4.2915	0.22356	-	-	-	3/28	9.786	0.33734
3	Glycine, serine and threonine metabolism	2/34	3.918	0.20661	-	-	-	2/34	5.3511	0.2066
4	Glycerophospholipid metabolism	2/36	3.8091	0.07396	1/361	1.8573	0.04814	1/36	2.1708	0.0258
5	Aminoacyl-tRNA biosynthesis	2/48	3.2693	0.16667	-	-	-	3/48	8.1425	0.16667
6	Thiamine metabolism	1/7	3.0867	0.0	-	-	-	-	-	-
7	Arginine biosynthesis	1/14	2.4144	0.0	-	-	-	2/14	7.1432	0.0
8	Nicotinate and nicotinamide metabolism	1/15	2.3483	0.0	1/15	2.6911	0.23465	1/15	3.0183	0.0
9	Histidine metabolism	1/16	2.2868	0.0	-	-	-	1/16	2.9551	0.0
10	Ether lipid metabolism	1/20	2.0755	0.0	1/20	2.4134	0.0	-	-	-
11	beta-Alanine metabolism	1/21	2.0297	0.0	-	-	-	1/21	2.6898	0.0
12	Sphingolipid metabolism	1/21	2.0297	0.0	-	-	-	1/21	2.6898	0.0
13	Glyoxylate and dicarboxylate metabolism	1/32	1.6409	0.04233	-	-	-	2/32	5.4716	0.04233
14	Cysteine and methionine metabolism	1/33	1.6131	0.02184	-	-	-	1/33	2.2538	0.02184
15	Purine metabolism	1/66	1.0159	0.05872	-	-	-	1/66	1.6045	0.0
16	Glutathione metabolism	-	-	-	2/28	5.019	0,28294	-	-	-
17	Pyrimidine metabolism	-	-	-	-	-	-	1/39	2.0947	0.0
18	Nitrogen metabolism	-	-	-	-	-	-	1/6	3.9227	0.0
19	D-glutamine and D-glutamate metabolism	-	-	-	-	-	-	1/6	3.9227	0.0

## 4 Discussion

Oxidative stress is considered an pivotal factor for cardiovascular diseases development, such as cardiac hypertrophy (Rababa'h et al., 2018). In this study, oxidative stress simulated by the use of DOX, ISO and PER in H9c2 cardiomyocyte cells caused a series of changes in several metabolic pathways. However, the supernatant samples obtained from the cell culture medium subjected to DOX, PER and ISO treatments did not display clear differences when compared with the control group. This information generated two hypotheses: 1) the excreted metabolites may not be significant for group discrimination; 2) the compounds that may be important for discriminating the studied groups were not extracted by the methodology used and/or analyzed by CE-MS. Therefore, a definitive answer about these hypotheses requires further sample exploration, such as using other extraction solventes or other analytical techniques.

Increased levels of some amino acids in the intracellular extracts of DOX- and PER-treated cells were identified. In both cases, serine and aspartic acid are notable. It is important to highlight that several amino acids can exist as D and L isomers due to the presence of a chiral center. For many years, only the L isomers of these amino acids were considered to be naturally present in mammalian tissues (MacKay et al., 2019). However, in the 1990’s, free D-serine was detected in the mammalian brain, followed by D-aspartate (Hadhimoto and Oka, 1997). On the other hand, Koneg and col. described that L-serine can be derived from four pathways, namely from a dietary source, from the biosynthesis of the glycolytic intermediate 3-phosphoglycerate, from glycine, and from the degradation of proteins and phospholipids (De Koning et al., 2003). However, Snell explains that enzymes from the serine biosynthetic pathway are present displaying high activity in the kidneys, testicles, spleen and brain, while presenting low activities in the skeletal and cardiac muscle (Snell, 1984). Thus, it is likely that the increased serine levels observed herein are due to protein and phospholipid degradation to obtain cell energy. Additionally, amino acids can be used as an alternative source of energy in some cases, as well as transformed into intermediates in the glycolysis and the tricarboxylic acid (TCA) cycle, such as phosphoenolpyruvate and acetyl-CoA (McNulty et al., 2000).

In this study, aspartic acid exhibited the highest levels in both the DOX and PER groups (FC = 141.14 and 95.92%, respectively). Aspartic acid is a non-essential amino acid naturally synthesized by mammals, which exists in two enantiomeric forms. The L-aspartate form is used in protein biosynthesis and neurotransmission (Johnson, 2017), and originates from the transamination of oxaloacetate, a TCA cycle intermediate (Bender, 2012a). In a recent study, Ritterhoff and col. demonstrated that increased glucose consumption is required to support aspartate synthesis that drives the biomass increases during cardiac hypertrophy (Ritterhoff et al., 2020). In mitochondria, it can be converted to oxaloacetate, which is a Krebs cycle (or TCA) substrate used in the production of ATP (Sivakumar et al., 2011). In the cytosol, L-aspartate is used in arginine biosynthesis, in the urea cycle (Liu et al., 2014). In turn, L-arginine is a substrate for nitric oxide synthase (NOS), responsible for the generation of nitric acid in endothelial cells, which is essential for proper cardiovascular system functioning (Lin et al., 2008).

Glutamine level changes were only observed in the group of cells subjected to PER treatment. Glutamine is a non-essential amino acid involved in the synthesis of cytoplasmic proteins and nucleotides. Its increment may be associated with augmented glutamine synthetase activity, an enzyme responsible for glutamine production from glutamate (Cruzat et al., 2018). In addition, glutamate accepts free ammonia from protein catabolism processes in muscle and peripheral tissues, forming glutamine (Häberle et al., 2018). Qi and col. mentioned that high PER concentrations (≥100 μmol L^−1^) cause the inhibition of α-ketoglutarate dehydrogenase in the TCA cycle. This enzyme is responsible for the conversion of α-ketoglutarate (a TCA cycle intermediate), coenzyme A and NAD^+^ to succinylCoA, NADH and CO_2_ (Qi et al., 2011). Oxoglutarate (also named α-ketoglutarate), can be modified to glutamate and, subsequently, glutamine (Bender, 2012b).

Choline also exhibited significant differences in both the DOX and PER groups. This metabolite is involved in several physiological processes, such as cholinergic neurotransmission and methylation, as the main source of methyl groups from trimethylglycine metabolite (Wortmann and Mayr, 2019). However, in this case, its participation as a precursor for phospholipid membrane synthesis, including phosphatidylcholine (PC), through the Kennedy pathway, is noteworthy. Phosphatidylcholine represents about 50% of the phospholipids in mammalian membranes and, therefore, acts in signaling and membrane transport (Zeisel, 2006; Gibellini and Smith, 2010; Sonkar et al., 2019). Thus, it is assumed that this metabolic alteration is due to the degradation of biological membranes in response to the oxidative stress caused by DOX and PER. In addition, it is important to note that membrane phospholipids are one of the main peroxidation targets due to the action of free radical (Phaniendra et al., 2015).

Still concerning the DOX treatment, decreases in the metabolic levels of certain vitamins, such as thiamine (vitamin B1) and pantothenic acid (vitamin B5), were remarkable. In this sense, oxidative stress and its damage are characterized by reduced levels of tissue antioxidants (Repetto et al., 2010). Vitamins are part of the non-enzymatic antioxidant defense system and act in inhibiting and/or reducing the damage caused by the harmful action of free radicals. In addition, pantothenic acid is a precursor to coenzyme A (CoA), which is essential in carbohydrate, lipid, and protein metabolism. An example of this situation is the conversion of pyruvate to acetyl-CoA for Krebs cycle participation (Gropper and Smith, 2012). Yi and col. in 2018, also reported decreased levels of pantothenic acid in H9c2 cells following DOX treatment (Yi et al., 2018).

Thiamine acts as a coenzyme in energy transformation, as well as in the synthesis of pentoses, nicotinamide, and adenine dinucleotide phosphate (NADPH). In addition, it acts as a non-coenzyme in nerve impulse transmission, and is extremely important in the activity of four cellular metabolic enzymes, pyruvate dehydrogenase (PDH) and alpha-ketoglutarate dehydrogenase (α-KGDH) in tricarboxylic acid (TCA), transketolase (TKT) within the pentose via phosphate (PPP), and the branched-chain alpha-keto-acid dehydrogenase complex (BCKDC) involved in amino acid catabolism. Alongside pantothenic acid and other B vitamins, it acts on the oxidative decarboxylation of pyruvate (Gropper and Smith, 2012).

The DOX group also exhibited an increase in adenosine monophosphate (AMP) levels. The increased AMP/ATP ratio activates the AMP-activated protein kinase (AMPK). This enzyme is responsible for cellular energy homeostasis. When activated, its function is to shut down the metabolic pathways that consume ATP, i.e., the synthesis of fatty acids and proteins, and stimulate the pathways that produce ATP, such as glycolysis and beta-oxidation. A study by Hinchy et al. indicated that ROS activates AMPK (Hinchy et al., 2018), strengthens the hypothesis that DOX treatment increased AMP levels and may activate AMPK.

N-acetylspermidine and glycerophosphocholine also displayed elevated levels following the use of DOX and ISO. The former is derived from the catabolism of spermidine, a natural polyamine that occurs in all living cells, which may be found as N1-acetylspermidine or N8-acetylspermidine, depending on the carbon chain adjacent to nitrogen in the acetylation process. Spermidine N1-acetyltransferase is the enzyme responsible for the conversion of N1-acetylspermidine, which is induced by high levels of polyamines (Giordano et al., 2010; Moriya et al., 2012; Nayak et al., 2020). Polyamines (putrescine, spermidine and spermine) and their derivatives are essential for cardiac cell proliferation, differentiation and protein synthesis (Meana et al., 2016). However, growing evidence indicates that they may also be involved in apoptosis and cell autophagy (Teringova and Tousek, 2017; Thomas and Thomas, 2001; Gustafsson and Gottlieb, 2009). Recently, a study conducted by Nayak and collaborators demonstrated that high levels of N8-acetylspermidine are associated with higher mortality in patients with ischemic cardiomyopathy (ICM) (Nayak et al., 2020). Therefore, in this case, N8-acetylspermidine can be considered a mechanistic ICM biomarker. In addition, Tantini et al. reported the involvement of polyamines in apoptosis of H9c2 cardiomyoblasts in a model of simulated ischemia (Tantini et al., 2006).

Glycerophosphocholine arises from a catabolic path following the breakdown of phosphatidylcholine, later converted into free choline (Bender, 2012b). As stated earlier, choline is a precursor for phospholipid membrane synthesis. Thus, it can be considered that this metabolic alteration supports the requirement for biological membrane turnover in response to oxidative stress caused by DOX and ISO. In 2020, Dallons and col. reported an increase in glycerophosphocholine levels following H9c2 cells exposure for 24 h to doxorubicin (0.3 μmol L^−1^) (Dallons et al., 2020), which agrees with the results presented herein.

Glutathione is a tripeptide found essentially in all aerobic organisms, and was found in higher levels in the ISO group. This compound plays an important role in the biotransformation and elimination of xenobiotics and in cell defense against oxidative stress. In addition, it can exist in both its reduced (GSH) or oxidized (GSSG) form, interconverted by glutathione reductase and glutathione peroxidase (Meister, 1983). Herein, increased GSSG and GSH levels were detected, suggesting possible glutathione reductase and glutathione peroxidase inhibition, resulting in the accumulation of these substances in the intracellular environment. Increased GSSG levels were also observed by Liu and colleagues, who concluded that isoproterenol can inhibit both enzymes in the heart, leading to GSSG accumulation (Liu et al., 2013). However, Sun et al. demonstrated the depletion of glutathione levels as an isoproterenol effect in rat hearts, in disagreement with was observed in the present study (Sun et al., 2017). Furthermore, the average ratio between reduced glutathione and oxidized glutathione (GSH/GSSG) under normal conditions, is 10:1 or greater, depending on the cell type, and an altered ratio is indicative of oxidative stress. In addition, glutathione reductase activity depends on adequate levels of the reduced forms of nicotinamide and adenine dinucleotide phosphate (NADPH) (Meister, 1983). However, GSH and GSSG measurement, in particular for the calculation of the GSH/GSSG ratio, is hampered by many artifacts (Giustarini et al., 2016). Particularly, the determination of GSH in biological fluids is a challenge due to GSH self-oxidation to GSSG, which results in lower GSH/GSSG ratios, in addition to the proteolytic degradation of both GSH and GSSG by γ-glutamyl transferase (Olmos Moya et al., 2017; Hamad et al., 2020).

Cells treated with ISO also presented increased S-hydroxymethyl-glutathione levels. This metabolite is the product of the reaction between formaldehyde and glutathione and is one of the main substrates for S-nitrosoglutathione reductase (GSNO-R), an enzyme that regulates S-nitrosation (SNO) and protects against formaldehyde toxicity (Lu et al., 2009; Casin and Kohr, 2020). Several *in vitro* studies have clearly indicated that formaldehyde is genotoxic (Speit and Schmid, 2006). On the other hand, S-nitrosation involves cysteine oxidation and corresponds to a signaling pathway for nitric oxide (NO), which is considered a cardiovascular homeostasis regulator, promoting vasorelaxation (Casin and Kohr, 2020). In this sense, NO reacts with glutathione to form S-nitrosoglutathione (GSNO). Hogg highlighted that cardiomyocytes employ GSNO as a physiological reservoir for NO (Hogg, 1999). Furthermore, GSNO and other nitrosating agents are important for reducing ischemia-reperfusion injury (I/R), increasing SNO contents in key proteins (Kohr et al., 2011; Sun et al., 2012; Huang et al., 2013; Tong et al., 2014; Sun et al., 2015; Zhang et al., 2016). Interestingly S-hydroxymethyl-glutathione is oxidized via electron transfer to NAD^+^, producing S-formylglutathione, which is then hydrolyzed to glutathione and formate (Sanghani et al., 2003; Casin and Kohr, 2020).

Consistently, NAD^+^ was over-expressed in the cell extracts obtained from H9c2 cells treated with ISO. NAD^+^ is an important coenzyme that acts in redox reactions in several pathways involved in energy production, such as glycolysis, the TCA cycle and fatty acid oxidation. NAD^+^ is usually reduced to NADH in these processes. However, the opposite (NADH oxidation to NAD^+^) may occur under anaerobic conditions, when pyruvate is reduced to lactate by lactate dehydrogenase - LDH (Kulkarni and Brookes, 2019; Tannous et al., 2020; Lin et al., 2021; Navas and Carnero, 2021). In addition, NAD^+^ acts as a substrate for several enzymes, such as poly (ADP-ribose) polymerase (PARP-1) and class III histone deacetylases (including the sirtuin family) (Hsu et al., 2009; Walker and Tian, 2018). PARPs act in DNA repair and sirtuin activity is linked to the metabolic state of cells (Houtkooper et al., 2010; Cantó and Auwerx, 2012).

In mammalian cells, NAD^+^ is produced mainly via the salvage pathway. Initially, nicotinamide mononucleotide (NMN) is formed from NAM and 5-phosphoribosyl-1-pyrophosphate (PRPP). This conversion is catalyzed by nicotinamide phosphoribosyltransferase (NAMPT). Then, NMN becomes conjugated to ATP and is transformed into NAD^+^ by NMN adenylyltransferase (NMNAT) (Revollo et al., 2004; Berger et al., 2005). It is known that cardiac stress alters the expression of the NAMPT protein and consequently, NAD^+^ levels (Hsu et al., 2014). Therefore, reduced NAD^+^ levels in cardiac tissue and in mitochondria were observed in acute ischemia (Di Lisa et al., 2001). Moreover, a study performed by Hsu and colleagues in 2009 on cardiomyocytes showed that NAMPT-specific cardiac overexpression increases NAD^+^ levels while protecting the heart from ischemia and reperfusion injury (Hsu et al., 2009). Authors suggest that NAMPT enzyme regulates the rate of NAD^+^ synthesis in cardiomyocytes. In this case, it is assumed that this NAD^+^ increase stimulates autophagy to improve protein quality control and increase ATP levels (Hsu et al., 2009). The NAD^+^ homeostasis process is also altered in cardiac hypertrophy. Studies employing mouse models induced to cardiac hypertrophy through Angiotensin II or isoproterenol have revealed an association of hypertrophy with intracellular NAD^+^ losses (Pillai et al., 2010). Finally, NAD^+^ and NAMPT level alterations comprise a consensus in certain pathological conditions. Future follow-up studies performed in hearts of experimental animals could confirm the findings of this study.

## 5 Conclusion

In the present study CE-MS technique was applied to analyze the intra and extracellular metabolome of differentiated H9c2 cardiomyocyte. The aim of this work was the analyses of possible alterations in cellular metabolism when simulating an oxidative stress through different ways, using hydrogen peroxide (PER), and the drugs doxorubicin (DOX) and isoproterenol (ISO). Our results showed that the intracellular content presented significant changes between the studied groups, but the extracellular did not. Amino acids, vitamins, tripeptides, and polyamines are within the altered metabolites, therefore, presenting changes mainly in energy metabolism, antioxidant pathways, and cell viability. These data showed that, despite the use of oxidative stress induction, each one generated different results in the cells and that it was possible to create a model to differentiate them, proving that the use of metabolomics is a useful tool for this purpose. Since only one analytical technique was used, herein further research is required to broaden comprehension on the investigated biological systems, pointing to multiplataform metabolomics as a future perspective.

## Data Availability

The raw data supporting the conclusions of this article will be made available by the authors, without undue reservation.
