# Supplementary material for: Untargeted Metabolomics Studies of H9c2 Cardiac Cells Submitted to Oxidative Stress, β-Adrenergic Stimulation and Doxorubicin Treatment: Investigation of Cardiac Biomarkers
Source: Front Mol Biosci. 2022 Jun 29;9:898742. doi: 10.3389/fmolb.2022.898742 (PMC9277393; doi:10.3389/fmolb.2022.898742)
Supplement: Supplementary file 1 [file DataSheet1.pdf]

Supplementary Material

Untargeted Metabolomics Studies of H9c2 Cardiac Cells Submitted to Oxidative Stress, β-Adrenergic Stimulation and Doxorubicin Treatment: Investigation of Cardiac Biomarkers

Monica Força Lima[1,2], Alan Gonçalves Amaral[2], Isabela Aparecida Moretto[3], Franckson Jhonne Torres Neves Paiva da Silva[3], Flávia Oliveira Borges Pereira[3], Coral Barbas[1], Aline Mara dos Santos[3], Ana Valéria Colnaghi Simionato[2,4], Francisco Javier Rupérez[1]*

[1] Center for Metabolomics and Bioanalysis (CEMBIO), Facultad de Farmacia, Universidad San Pablo-CEU, CEU Universities, Boadilla del Monte, 28660, Madrid, Spain;

[2] Department of Analytical Chemistry, Institute of Chemistry, University of Campinas, 13083-970, Campinas - SP, Brazil;

[3] Department of Structural and Functional Biology, Institute of Biology, University of Campinas, 13083-970, Campinas - SP, Brazil;

[4] National Institute of Science and Technology in Bioanalytics (INCTBio).

* Corresponding authors: e-mail adress: ruperez@ceu.es (Francisco Javier Rupérez) and alinems@unicamp.br (Aline Mara dos Santos)

1 Supplementary Figures

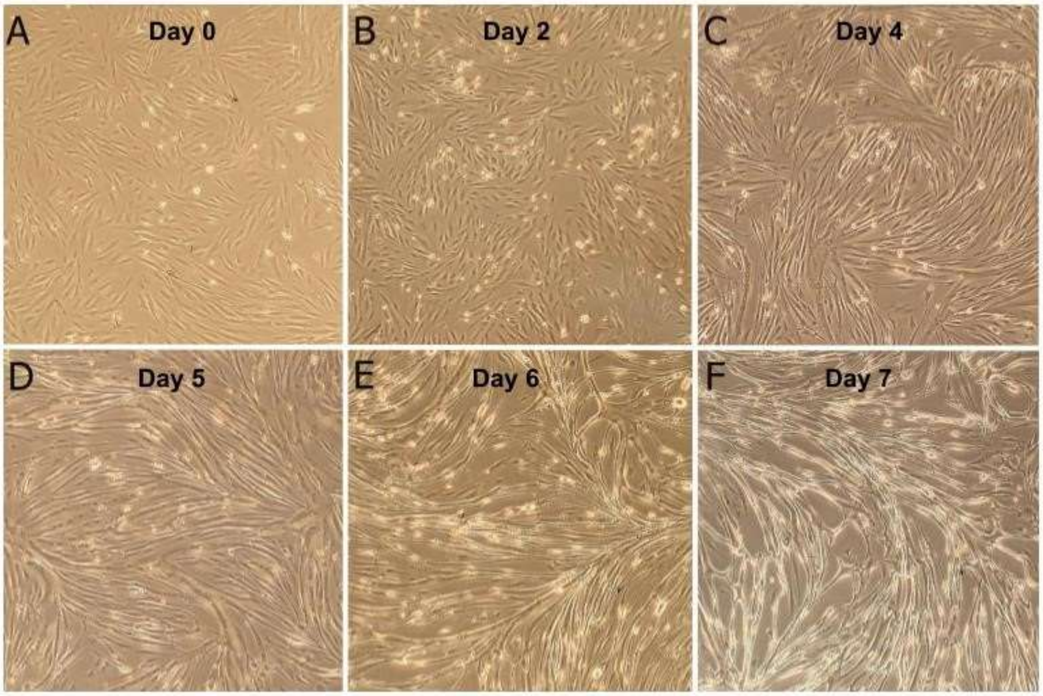

Supplementary Figure 1: Phase-contrast images from H9c2 cells during the differentiation process. The images were captured with a 100x magnificence, during 7 consecutive days as indicated in the sequence from A to F. The differentiated H9c2 cardiomyocytes are morphologically elongated and multinucleated, while the undifferentiated cells have a polygonal shape.

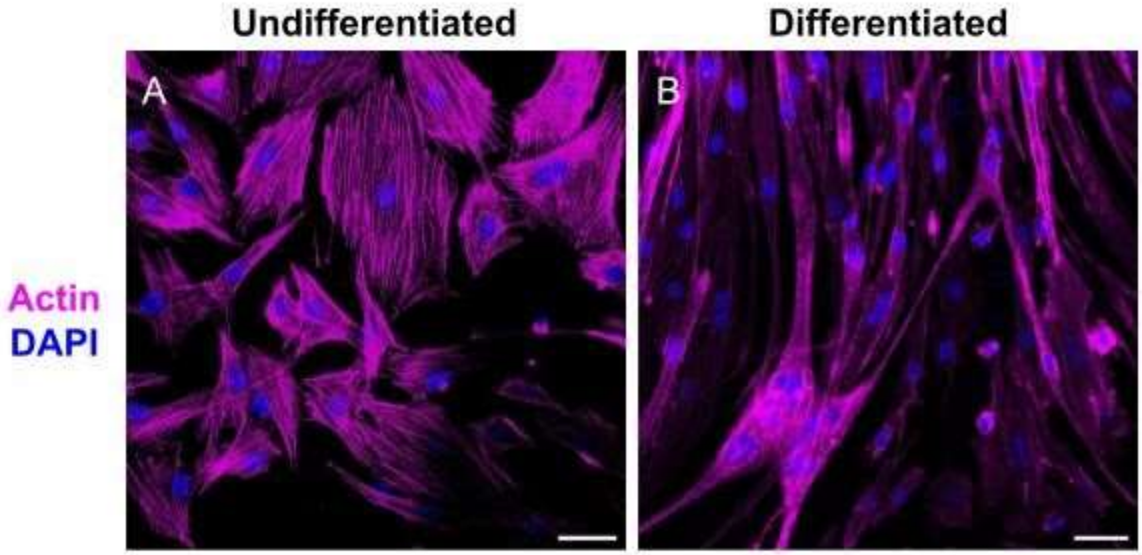

Supplementary Figure 2: Immunofluorescence images of H9c2 cardiomyocytes. A. Super resolution image of undifferentiated myocytes shows evenly distributed polygonal shaped cells. B. After differentiation, myocytes became multinucleated and elongated. Magenta: actin; Blue: core. Scale bar = 50 µm.

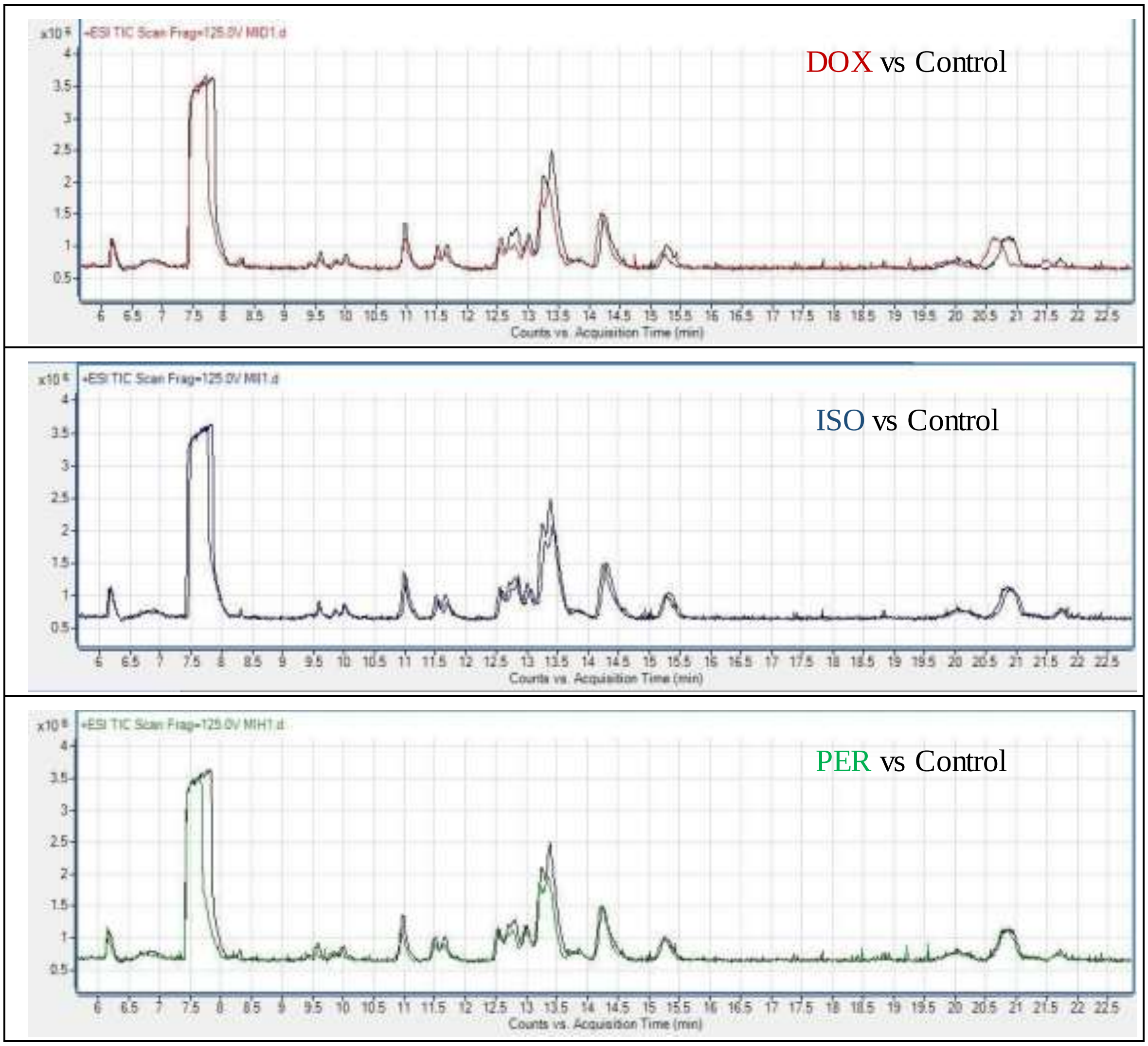

Supplementary Figure 3. Total ion electropherogram (TIE) obtained from the cellular extract of H9c2 cells that were submitted to DOX, ISO, and PER and analyzed by CE-MS in the positive ionization mode.

DOX vs Control

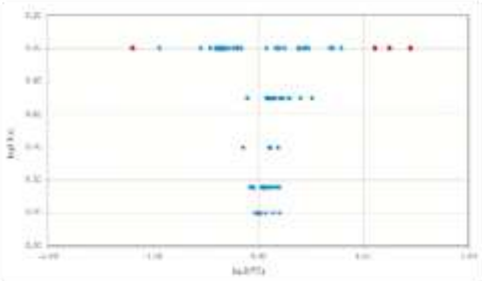

Features

103.0647@10.32	257.1035@20.77	187.1678@8.90	133.0372@13.87
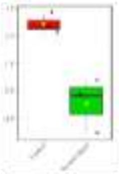	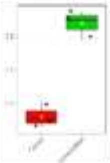	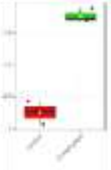	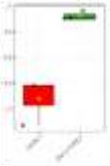

PER vs Control

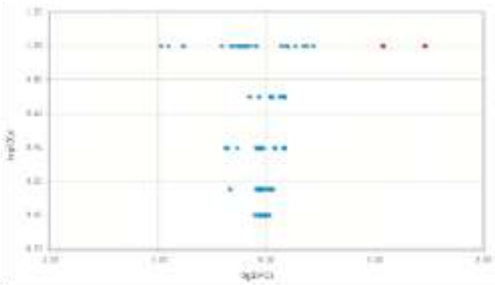

Features

105.0428@12.53	133.0372@13.87
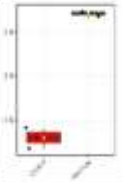	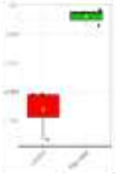

Supplementary Figure 4: Important molecular features selected by volcano plot with fold change threshold (x) 2 and t-tests threshold (y) 0.1 obtained from H9c2 cell extract in the untargeted metabolomic evaluation by CE-MS carried out in the positive ionization mode.

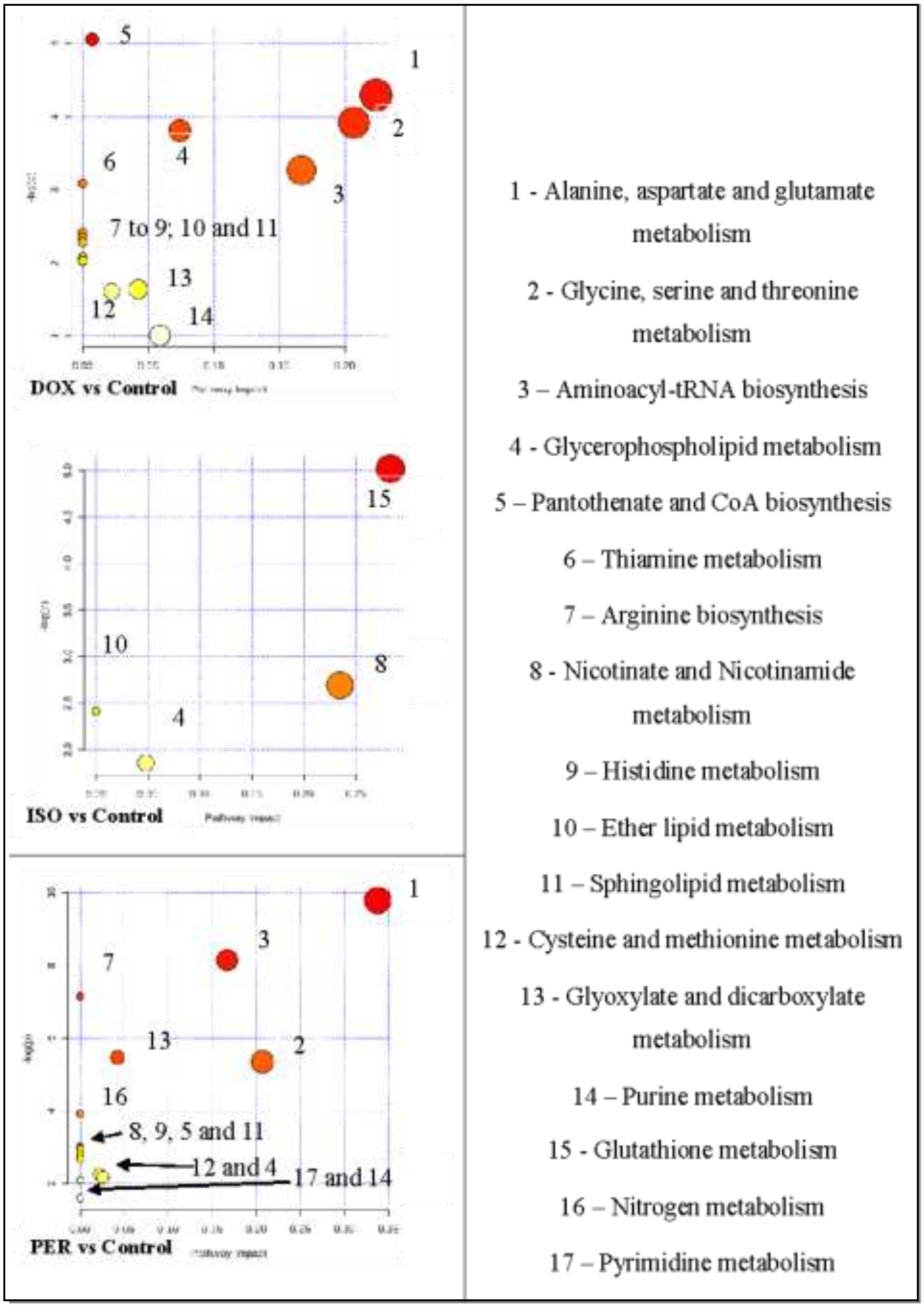

Supplementary Figure 5: Pathway analysis for the comparison of the three treatments applied to H9c2 cells. Annotations: Pathway analysis carried out in MetaboAnalyst 4.0, Pathway library Mus musculus (KEGG), Pathway analysis algorithms were “Hypergeometric Test” for Over Representation Analysis and “Relative-betweeness Centrality” for Pathway Topology Analysis.
